# Multiple resiliency metrics reveal complementary drivers of ecosystem persistence: An application to kelp forest systems

**DOI:** 10.1002/ecy.4453

**Published:** 2024-10-27

**Authors:** Jorge Arroyo‐Esquivel, Riley Adams, Sarah Gravem, Ross Whippo, Zachary Randell, Jason Hodin, Aaron W. E. Galloway, Brian Gaylord, Marissa L. Baskett

**Affiliations:** ^1^ Department of Mathematics University of California Davis California USA; ^2^ California Department of Fish and Wildlife West Sacramento California USA; ^3^ Department of Environmental Science and Policy University of California Davis California USA; ^4^ Department of Integrative Biology Oregon State University Corvallis Oregon USA; ^5^ Department of Biology University of Oregon Charleston Oregon USA; ^6^ Seattle Aquarium Seattle Washington USA; ^7^ Friday Harbor Labs University of Washington Friday Harbor Washington USA; ^8^ Bodega Marine Laboratory University of California Davis California USA; ^9^ Department of Evolution and Ecology University of California Davis California USA

**Keywords:** disturbance, kelp forest, resiliency metrics, restoration, trophic model

## Abstract

Human‐caused global change produces biotic and abiotic conditions that increase the uncertainty and risk of failure of restoration efforts. A focus of managing for resiliency, that is, the ability of the system to respond to disturbance, has the potential to reduce this uncertainty and risk. However, identifying what drives resiliency might depend on how one measures it. An example of a system where identifying how the drivers of different aspects of resiliency can inform restoration under climate change is the northern coast of California, where kelp experienced a decline in coverage of over 95% due to the combination of an intense marine heat wave and the functional extinction of the primary predator of the kelp‐grazing purple sea urchin, the sunflower sea star. Although restoration efforts focused on urchin removal and kelp reintroduction in this system are ongoing, the question of how to increase the resiliency of this system to future marine heat waves remains open. In this paper, we introduce a dynamical model that describes a tritrophic food chain of kelp, purple urchins, and a purple urchin predator such as the sunflower sea star. We run a global sensitivity analysis of three different resiliency metrics (recovery likelihood, recovery rate, and resistance to disturbance) of the kelp forest to identify their ecological drivers. We find that each metric depends the most on a unique set of drivers: Recovery likelihood depends the most on live and drift kelp production, recovery rate depends the most on urchin production and feedbacks that determine urchin grazing on live kelp, and resistance depends the most on feedbacks that determine predator consumption of urchins. Therefore, an understanding of the potential role of predator reintroduction or recovery in kelp systems relies on a comprehensive approach to measuring resiliency.

## INTRODUCTION

The ecological disturbance produced by human‐caused global change challenges the traditional view of ecological management and restoration that targets historical representation. New climate regimes may produce conditions where restoring to a historical representation may not be possible (Harris et al., 2006). In addition to changes in abiotic conditions, population extinctions and invasive species can lead to novel, unobserved ecosystem states that might cause managers to set new ecosystem‐level goals for these new states (Hobbs et al., 2009). These changes increase the uncertainty of the fate of ecosystems, which makes the decision‐making process more challenging overall (Polasky et al., 2011). To address these challenges, a growing literature has proposed a focus of management goals on ecological resiliency, that is, the ability of the system to respond to disturbance (Chapin et al., 2010; Millar et al., 2007; Spears et al., 2015). Achieving this goal of managing for ecological resiliency relies on an understanding of the ecological drivers of this resiliency (Scheffer & Carpenter, 2003).

Identifying drivers of resiliency can depend on how one measures it (Donohue et al., 2016). One metric of resiliency is the recovery likelihood, which is the likelihood of maintaining a target, undisturbed state (Holling, 1973). A second, another commonly used metric is the rate at which the ecosystem returns to an undisturbed state after disturbance (Holling, 1996). The third metric is resistance to disturbance, that is, the amount that a system changes for a given level of disturbance (Nolting & Abbott, 2016). If alternative stable states are relevant to the system (i.e., multiple states are possible under the same environmental conditions), then these metrics can be measured by the size of the basin of attraction of the state of interest for recovery likelihood, how long it takes to move from one state to the other after disturbance for the recovery rate (Arani et al., 2021), or how much disturbance is required to move from one state to the other for resistance (Nolting & Abbott, 2016). Resilience in general depends on an array of drivers such as diversity, functional redundancy, modularity, and the tightness of feedback loops (Ives & Carpenter, 2007; Levin & Lubchenco, 2008; Steiner et al., 2006; Walker, 1995). Resolving how different resiliency metrics depend on different drivers and their associate processes can then inform a comprehensive approach to managing for multiple aspects of resiliency by targeting an array of complementary drivers and ecological processes. Given multiple resilience metrics, a question is then whether or not they differ in their dependence on different drivers (Ingrisch & Bahn, 2018; Quinlan et al., 2016).

Feedback loops and their effect on resiliency depend on an array of community processes (Folke et al., 2004). In particular, feedback loops can arise from either consumptive effects (CEs) or nonconsumptive effects (NCEs) that alter consumption through behavioral or other modifications. CEs affect the strength of species interactions that determine how a disturbance might cascade through a system by reducing the density of specific populations through consumption. An example of this in a three‐level system would be a disturbance‐driven decline in top predators, leading prey to overconsume basal resources (Rudman et al., 2016). NCEs can change the strength of CEs and therefore change the effect of disturbance cascading through a system. For example, disease transmission between trophic levels or fear of predation by herbivores can suppress the CEs of its trophic level and therefore overconsumption, which can lead to a more resilient system (Bestion et al., 2015; Sharp & Angelini, 2021).

The Northeast Pacific kelp system provides a useful example for identifying how the drivers of different aspects of resiliency can inform management under climate change. This part of the coast experienced a severe marine heat wave, where increased water temperatures negatively impacted the growth rate of kelp while increasing the grazing rates of the purple sea urchin *Strongylocentrotus purpuratus* (Murie & Bourdeau, 2021; Simonson et al., 2015). Furthermore, the functional extinction of a primary predator of the purple urchins, the sunflower sea star *Pycnopodia helianthoides*, on the California coast due to the sea star wasting disease released urchins from predation, which led to an overall increased consumption of kelp (Hamilton et al., 2021; Rogers‐Bennett & Catton, 2019). In addition to these cascading CEs, three NCEs affect grazing outcomes. First, the presence of cues of some predators such as the spiny lobster in Southern California (Matassa, 2010) and sunflower sea stars (Freeman, 2006) induces a fear effect on sea urchins, which reduces foraging activity to reduce predation risk. Second, sea urchins exhibit a preference for drift kelp (pieces of kelp that break off and drift to the sea floor) over live kelp, where sea urchin direct grazing pressure on extant kelp populations is greater when kelp density is too low to produce sufficient drift kelp (Kriegisch et al., 2019; Randell et al., 2022). Third, at low kelp densities, starved urchins with poor nutritional condition are consumed at higher rates by *Pycnopodia* than well‐fed urchins in kelp forests (Galloway et al., 2023). Through this combination of events, CEs, and NCEs, kelp coverage on the northern coast of California has declined by over 95% (McPherson et al., 2021; Rogers‐Bennett & Catton, 2019).

Restoration efforts focused on urchin removal and kelp reintroduction are underway. These restoration efforts have the potential to enhance near‐term kelp recovery (Arroyo‐Esquivel et al., 2023; Ward et al., 2022). However, in the longer term, climate might increase disturbance through increased marine heat wave intensity or severity (Prochaska et al., 2023) or disease severity (Scavia et al., 2002). This raises the question of how current and possible further restoration efforts (e.g., the reintroduction of a sea urchin predator) might affect the resiliency of the kelp forest to future disturbance.

Here, we quantify how different metrics of resiliency are affected by different ecosystem processes, using the Northern California kelp forest as an example system. To do this, we build a kelp–urchin–predator food chain model, described in detail in the *Model* section, that incorporates the NCE feedbacks of predator fear response by urchins to predatory sea stars, drift kelp preference by urchins, and starvation‐dependent predation susceptibility of sea urchins. We then use this model to analyze how the different ecological processes included in this model affect three resiliency metrics: recovery likelihood, recovery rate, and resistance to disturbance to a kelp forest state. Finally, we consider how these ecological processes might connect to restoration interventions such as future consideration of the reintroduction of an urchin predator.

## METHODS

### Model

**FIGURE 1:**
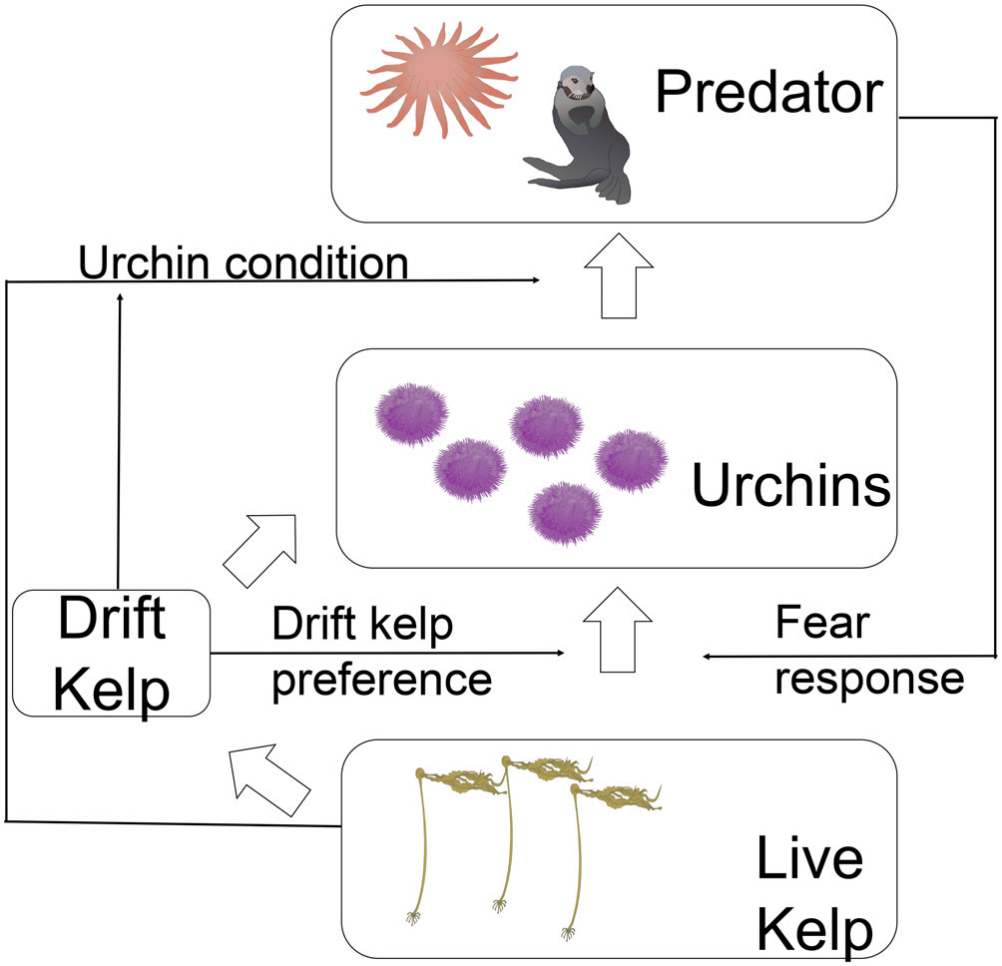
Overview of the model dynamics. The model describes the dynamics of a tritrophic food chain, where the resource (kelp) is divided into two groups: live kelp and drift kelp. The open arrows represent the energy flows from one stage of the food chain to the next one (consumptive effects [CEs]), whereas the solid black arrows represent nonconsumptive effects (NCEs) that modify these consumption rates, and the origin of the arrow describes which density affects the indicated consumption rate. Diagram images thanks to Jane Thomas, IAN Image Library (https://ian.umces.edu/imagelibrary/) under license CC BY‐SA 4.0.

Our model follows the growth rates for densities of live kelp A, drift kelp D, urchin U, and a predator S through time (Figure 1). Throughout this paper, we base the predator dynamics and parameters on sunflower sea stars. In the *Discussion* section, we also discuss how our model outcomes might relate to those in the case wherein the predator was the sea otter *Enhydra lutris*, which has been locally extinct on the California north coast since the mid‐1800s (Lubina & Levin, 1988). Our model describes the interactions between live kelp, urchin, and predator densities based on a Rosenzweig–MacArthur three‐species food chain (Rosenzweig & MacArthur, 1963). We incorporate NCEs by multiplying the functional forms of consumption by a factor dependent on a specific population density. We model the fear response of urchins to predators and drift kelp preference using the functional form for fear factor described by Sasmal and Takeuchi (2020), whereas the starvation‐dependent predation susceptibility of urchins is a function of kelp with a factor that modifies the predator predation rate on sea urchins.

We assume that live kelp density follows a logistic growth model with a growth rate r and a carrying capacity K. To account for the urchin preference for drift kelp (Kriegisch et al., 2019), we assume that kelp active grazing by urchins follows a Type I (linear) Holling functional response with a baseline grazing rate $\alpha_{A}$ multiplied by a decreasing factor of drift kelp density. In particular, we assume that the proportion of total grazing time that urchins graze on live kelp A is inversely related to drift kelp availability D modified by a factor $\kappa_{D}$, which modulates the strength of drift kelp preference; urchins spend the remaining proportion of the total grazing time passively subsisting on drift kelp with a cryptic behavior (e.g., hiding in crevices). In addition, we incorporate fear of predation by reducing the active grazing rate by a factor depending on predator density with a fear parameter $\kappa_{S}$. Finally, we assume that live kelp biomass breaks off into drift kelp following a density‐independent rate $\delta_{A}$.

To model the rate of change in drift kelp density, we assume that a fraction $\varepsilon_{D}$ of the live kelp biomass that breaks off into drift kelp is retained by the system. This drift kelp is then consumed by urchins following a Type I Holling functional response with a baseline consumption rate $\alpha_{D}$ multiplied by an increasing factor of drift kelp density based on the proportional distribution of grazing time with the modifying factor $\kappa_{D}$ described in the previous paragraph. Finally, we assume that drift kelp degrades at a density‐independent rate $\delta_{D}$.

Urchins reproduce based on energetic gain through consumption of kelp, from either direct active grazing or drift kelp consumption, with a conversion efficiency $\varepsilon_{U}$. Urchins are consumed by predators following a Type II (saturating) Holling functional response with a baseline predation rate $\alpha_{U}$ and a saturation parameter $\gamma_{U}$. Laboratory analyses show that sea stars consume more urchins when urchins are starving, likely due to a faster handling time (Galloway et al., 2023). We assume that urchin susceptibility to predation is directly linked to kelp density, where we model the predation of urchins as a decreasing function of kelp density for both live and drift kelp, similar to the fear of predations by urchins, with a parameter $\kappa_{A}$. Finally, we assume that urchins naturally die with a density‐independent death rate $\delta_{U}$.

Predators reproduce based on energetic gain through predation of urchins with a baseline conversion efficiency $\varepsilon_{S}$. Although sea stars consume fewer urchins when urchins are not starving (i.e., in a low predation susceptibility due to high kelp availability), non‐starving urchins are more nutritious due to a higher gonad content, which increases predator energetic gain (Murie & Bourdeau, 2021). We model this by increasing the conversion efficiency with kelp density by a factor β. Finally, predators naturally die with a density‐independent death rate $\delta_{S}$.

Our system dynamics are then

(1) $$\begin{array}{l} \frac{\mathrm{dS}}{\mathrm{dt}}=\frac{\varepsilon_{S}\left(1+\beta \left(A+D\right)\right)\alpha_{U}\mathrm{US}}{1+\gamma_{U}U}\frac{1}{1+\kappa_{A}\left(A+D\right)}-\delta_{S}S,\\ \frac{\mathrm{dU}}{\mathrm{dt}}=\frac{1}{1+\kappa_{D}D}\varepsilon_{U}\alpha_{A}\mathrm{AU}\frac{1}{1+\kappa_{S}S}+\left(1-\frac{1}{1+\kappa_{D}D}\right)\varepsilon_{U}\alpha_{D}\mathrm{DU}\\ \;-\frac{\alpha_{U}\mathrm{US}}{1+\gamma_{U}U}\frac{1}{1+\kappa_{A}\left(A+D\right)}-\delta_{U}U,\\ \frac{\mathrm{dD}}{\mathrm{dt}}=\varepsilon_{D}\delta_{A}A-\left(1-\frac{1}{1+\kappa_{D}D}\right)\alpha_{D}\mathrm{DU}-\delta_{D}D,\\ \frac{\mathrm{dA}}{\mathrm{dt}}=\mathrm{rA}\left(1-\frac{A}{K}\right)-\frac{1}{1+\kappa_{D}D}\alpha_{A}\mathrm{AU}\frac{1}{1+\kappa_{S}S}-\delta_{A}A. \end{array}$$

To simplify our analysis, we assume that drift kelp reaches equilibrium (i.e., drift kelp reaches a state in which its density does not change in time) faster than the other dynamics in the system. This allows us to use the simplifying assumption dD/dt=0 at a drift kelp density $D^{*}=D^{*}\left(A,U\right)$. This reduces our system to

(2) $$\begin{array}{l} \frac{\mathrm{dS}}{\mathrm{dt}}=\frac{\varepsilon_{S}\left(1+\beta \left(A+D^{*}\right)\right)\alpha_{U}\mathrm{US}}{1+\gamma_{U}U}\frac{1}{1+\kappa_{A}\left(A+D^{*}\right)}-\delta_{S}S,\\ \frac{\mathrm{dU}}{\mathrm{dt}}=\frac{1}{1+\kappa_{D}D^{*}}\varepsilon_{U}\alpha_{A}\mathrm{AU}\frac{1}{1+\kappa_{S}S}\\ \;+\left(1-\frac{1}{1+\kappa_{D}D^{*}}\right)\varepsilon_{U}\alpha_{D}D^{*}U\\ \;-\frac{\alpha_{U}\mathrm{US}}{1+\gamma_{U}U}\frac{1}{1+\kappa_{A}\left(A+D^{*}\right)}-\delta_{U}U,\\ \frac{\mathrm{dA}}{\mathrm{dt}}=\mathrm{rA}\left(1-\frac{A}{K}\right)-\frac{1}{1+\kappa_{D}D^{*}}\alpha_{A}\mathrm{AU}\frac{1}{1+\kappa_{S}S}-\delta_{A}A. \end{array}$$

### Parameter estimation

We numerically analyze System 2 using parameter values from a variety of sources for bull kelp (*Nereocystis luetkeana*) systems as our baselines (Table 1). For kelp growth dynamics, we use the growth factor, carrying capacity, and drift kelp production parameters of Arroyo‐Esquivel et al. (2023) as a baseline. To convert these parameters from kelp coverage to kelp biomass, we use a conversion factor of 1.3709 kg kelp/m^2^ per plant/m^2^ based on the data found by Stekoll et al. (2006). We then calibrate the parameters so that, in the absence of urchins, kelp reaches carrying capacity after a span of approximately 18 weeks as observed by Foreman (1984).

**TABLE 1:** Description of the parameters of our model, including the baseline values and the distribution of values explored in the global sensitivity analysis.

Parameter	Description	Baseline value	Distribution of values explored
r	Growth rate of kelp at low densities	2.5 g kelp (week)^−1^	$\text{Normal}\;\left(2.5,,\;,1.5\right)$
K	Carrying capacity of kelp	10,000 g kelp	$\text{Normal}\;\left(10,000,\;5000\right)$
$\kappa_{D}$	Effect of drift presence on the urchin grazing rate	1.95 g kelp^−1^	$\text{Bernoulli}\;\left(0.9\right)\times \text{Normal}\;\left(2,\;1\right)$
$\alpha_{A}$	Urchin grazing rate at low kelp densities	0.025 (urchins week)^−1^	$\text{LogNormal}\;\left(-3.7,\;1\right)$
$\kappa_{S}$	Effect of fear of predation on the urchin grazing rate	0.81 (sea stars)^−1^	$\text{Bernoulli}\;\left(0.9\right)\times \text{LogNormal}\;\left(-0.2,\;1\right)$
$\delta_{A}$	Conversion rate of live kelp biomass to drift kelp	1.8 week^−1^	$\text{LogUniform}\;\left(0.018,\;5\right)$
$\delta_{D}$	Drift kelp escape rate	0.3 week^−1^	$\text{LogUniform}\;\left(10^{-3},\;1\right)$
$\varepsilon_{D}$	Proportion of drift kelp retained in the system	0.7	$\text{Uniform}\;\left(0,\;1\right)$
$\varepsilon_{U}$	Conversion efficiency from grams of grazed kelp to urchins	0.1	$\text{LogUniform}\;\left(10^{-5},\;10^{-1}\right)$
$\alpha_{D}$	Drift kelp consumption rate by urchins	0.062 (urchins week)^−1^	$\text{LogNormal}\;\left(-2.7,\;1\right)$
$\alpha_{U}$	Predator predation rate at low urchin counts	4.77 (sea stars week)^−1^	$\text{Uniform}\;\left(0,10\right)$
$\gamma_{U}$	Predator predation saturation constant	3.42 (sea star)^−1^	$\text{LogUniform}\;\left(-2,\;3\right)$
$\kappa_{A}$	Starvation effect on urchin susceptibility to predation	0.00013 (g kelp)^−1^	$\text{Bernoulli}\;\left(0.9\right)\times \text{LogNormal}\;\left(-8,\;1\right)$
$\delta_{U}$	Natural death rate of urchins	0.0004 week^−1^	$\text{LogNormal}\;\left(-8,\;,-4\right)$
β	Impact of kelp density on nutritional value of urchins	0.1	$\text{Bernoulli}\;\left(0.9\right)\times \text{LogUniform}\;\left(-2,\;2\right)$
$\varepsilon_{S}$	Conversion proportion from urchins predated to predators	0.1	$\text{LogUniform}\;\left(10^{-5},\;10^{-1}\right)$
$\delta_{S}$	Natural death rate of predators	10^−4^ week^−1^	$\text{LogUniform}\;\left(-9,-2\right)$

For the parameters of drift kelp preference $\kappa_{D}$, the consumption rates of live kelp $\alpha_{A}$, and drift kelp $\alpha_{D}$, we estimate the parameters using the data of Randell (2021). For the proportion of drift kelp retained ε and escape rate $\delta_{D}$, we also use the parameters of Randell (2021).

We estimate the parameter values of predator dynamics based on data from experimental tests for the sunflower sea star. We determine the effect of fear of predation $\kappa_{S}$ by comparing the ratio of active grazing rates with and without sea stars with the ratio of sea stars to sea urchins used by Whippo et al. (2024) (1:7). The baseline parameters for urchin predation $\alpha_{U}$ and $\gamma_{U}$ and the effect of urchin starvation on predator predation $\kappa_{A}$ are based on the experimental tests of Galloway et al. (2023).

Finally, we estimate the death rate of the urchins using a baseline average lifespan of 50 years (Ebert, 2010). The parameters for conversion efficiencies of urchins $\varepsilon_{U}$ and the predator $\varepsilon_{S}$ and β, as well as the natural death rate of the predator $\delta_{S}$, were not possible to determine from the available data and are best guesses based on the expert opinion of the authors.

### Metrics of resiliency

**FIGURE 2:**
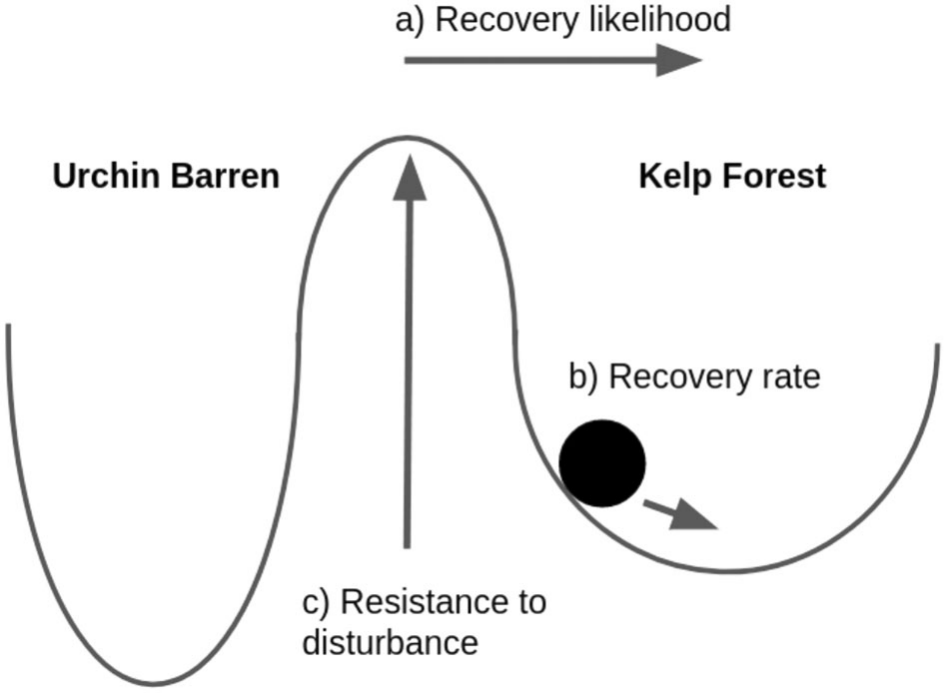
Ball‐and‐cup diagram that illustrates our resiliency metrics. Each cup is a possible system state, where the ball represents the present states as affected by disturbance on system dynamics, and the arrows represent the resiliency metrics: (a) the width of the cup to measure the recovery likelihood (basin of attraction to the target state), (b) steepness of the cup to measure the recovery rate (leading eigenvalue of the Jacobian of the system at the equilibrium for the target state), and (c) depth of the cup to measure resistance (amount of change expected per perturbation, i.e., quasipotential).

To quantify the resiliency of the kelp forest state, we use three different metrics. We illustrate these metrics with a ball‐and‐cup diagram, which is a heuristic visualization tool where the ball represents the current state of the system, and each cup represents an alternative stable state (Beisner et al., 2003) (see Figure 2). In this diagram, disturbances move the ball through the landscape of alternative stable states, where such movement can come from direct manipulation or through stochastic events (such as extreme heat waves or anomalous grazer or disease outbreaks). Resiliency metrics describe different components of a return to a basal state, determined by the cup in which it currently sits (target state or alternative stable state). The ball (the community state) transitions between states by moving past a hill through disturbance. How difficult it is to escape the cup after disturbance events depends on properties of the cup such as its width, steepness, and depth.

First, we measure the recovery likelihood (basin of attraction for the target state) as the proportion of initial community densities leading to the kelp forest state (Holling, 1973). We illustrate this metric in the ball‐and‐cup diagram (Figure 2) as the width of the cup, where a wider cup will include more possible states that eventually return to the kelp forest stable state. To measure this attribute, we create a grid of different kelp A, urchin U, and predator S initial population densities. For each combination of initial densities, we run our System 2 for 1000 weeks. Then, our recovery likelihood is the proportion of our grid of initial conditions where the final kelp density exceeds 95% of the equilibrium kelp density in the kelp forest stable state.

Second, we measure the recovery rate as the rate at which our system returns to the stable kelp forest state after a disturbance event. We illustrate this metric in the ball‐and‐cup diagram (Figure 2) with the steepness of the cup, where a steeper cup will lead to the ball returning faster to the bottom of the cup. This metric is the real part of the dominant eigenvalue of the Jacobian matrix of Model 2 evaluated at the kelp forest state (Neubert & Caswell, 1997).

Finally, the third metric for resiliency that we measure is the resistance of the kelp forest to disturbance. We illustrate this metric in the ball‐and‐cup diagram (Figure 2) as the depth of the cup, where it takes a larger disturbance to reach the top of a cup as the cup gets deeper (Nolting & Abbott, 2016). To quantify this resistance to stochasticity, we use the quasipotential (introduced in the context of ecology by Nolting & Abbott, 2016), which describes how much energy is required to transition from a community state to the other alternative stable state. Given that our system models more than two population densities, we estimate the quasipotential using the algorithm implemented in the Python package PyRitz (Kikuchi et al., 2020). This algorithm calculates the minimum energy required to transition from one stable state to another using classical mechanical principles. In this paper, we present the quasipotential going from the kelp forest state to the urchin barren state. Note that the quasipotential going from the urchin barren state to the kelp forest could be different and thus might have different drivers as those found in this paper.

Note that all three resilience metrics are agnostic of the source of disturbance; rather, they describe different aspects of the system response to any outside factor that has the potential to alter the state variables. For an application to kelp forest systems, we are particularly interested in disturbance caused by marine heat waves that might affect kelp and urchin densities (Murie & Bourdeau, 2021; Simonson et al., 2015) and disease outbreaks that might affect predator densities (Hamilton et al., 2021; Rogers‐Bennett & Catton, 2019). However, the resilience metrics can account for other possible sources of disturbance.

To explore the relative effect of each ecological process and account for the uncertainty of our parameter estimates, we run a global sensitivity analysis (GSA; described in Harper et al., 2011) for the three different metrics of resiliency as described above. The GSA algorithm consists of sampling the parameters of the model from a given distribution, which are then used to measure the different metrics of resiliency. The most important parameters that determine a given resiliency metric are then identified using the importance metric of a random forest analysis. The importance metric of a random forest is a relative measure of how much varying each individual parameter leads to a difference in the value of the specific metric of resiliency predicted by the trained random forest. This metric provides a relative comparison of parameter effects within each metric; absolute values are arbitrary and therefore cannot be compared across metrics. We sample parameters from empirical distributions determined by our parameter estimates and the confidence in these parameters. For the parameters with more certainty in their estimation, we use either Normal or LogNormal distributions, while we use Uniform or LogUniform distributions for less certain parameters. For the parameters related to NCEs ($\kappa_{D},\kappa_{S},\beta ,\kappa_{A}$), we oversample the value 0 to include simulations where certain NCEs are excluded; this lets our GSA measure the effect of the presence versus the absence of each NCE as well as the effect of its strength when present. To do this, we multiply the sampled numerical value of the NCEs by an independent Bernoulli distribution with a probability of 10% of being 0. The numerical values chosen for these empirical distributions are chosen close to the estimated values for more certain parameters and are chosen to cover a broad range of orders of magnitude for less uncertain parameters. These and the rest of the distributions used in the GSA are provided in Table 1. We also use the GSA to identify the top two most influential parameters for each resiliency metric for exploration in the local sensitivity analyses, with all the other parameters set to their baseline values, described in the section *Parameter estimation*. Notice that these local sensitivity analyses may not fully display the variability in these metrics, which are calculated by complex interactions of different parameters. However, these local sensitivity analyses provide a simple perspective on how varying one single parameter can affect the different resilience metrics. To preserve this simplicity, we present in the main text the single‐variable local sensitivity analysis and in Appendix S1 a more complex variation of parameters obtained from the parameters sampled for the GSA.

## RESULTS

**FIGURE 3:**
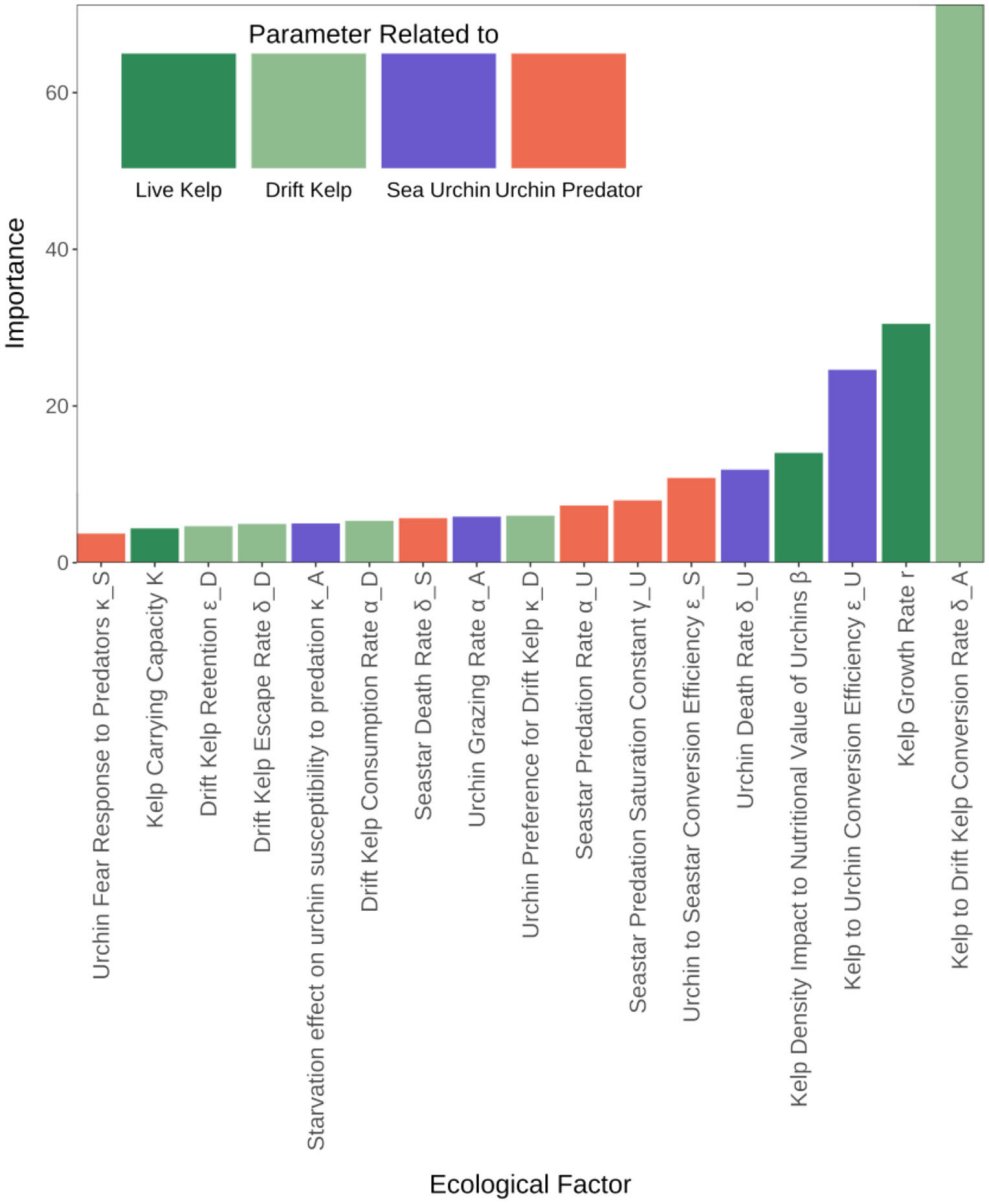
Importance ranking of the parameters of Model 2 from the global sensitivity analysis of the recovery likelihood of the kelp forest. See Table 1 for more detailed parameter definitions.

From our GSA, we find that each resiliency metric (recovery likelihood, recovery rate, and resistance) depends most on a different set of ecological drivers. The main drivers of recovery likelihood are drift production ($\delta_{A}$) and kelp growth (r) as well as, to a lesser extent than kelp growth, how the energy provided by kelp is transferred through the food chain (conversion efficiencies of kelp to urchin $\varepsilon_{U}$ and urchin to predator $\varepsilon_{S}$) (Figure 3). This suggests that the kelp forest is more likely to recover if kelp can either grow fast enough to survive active grazing or produce enough drift kelp that will deter urchins from actively grazing it.

**FIGURE 4:**
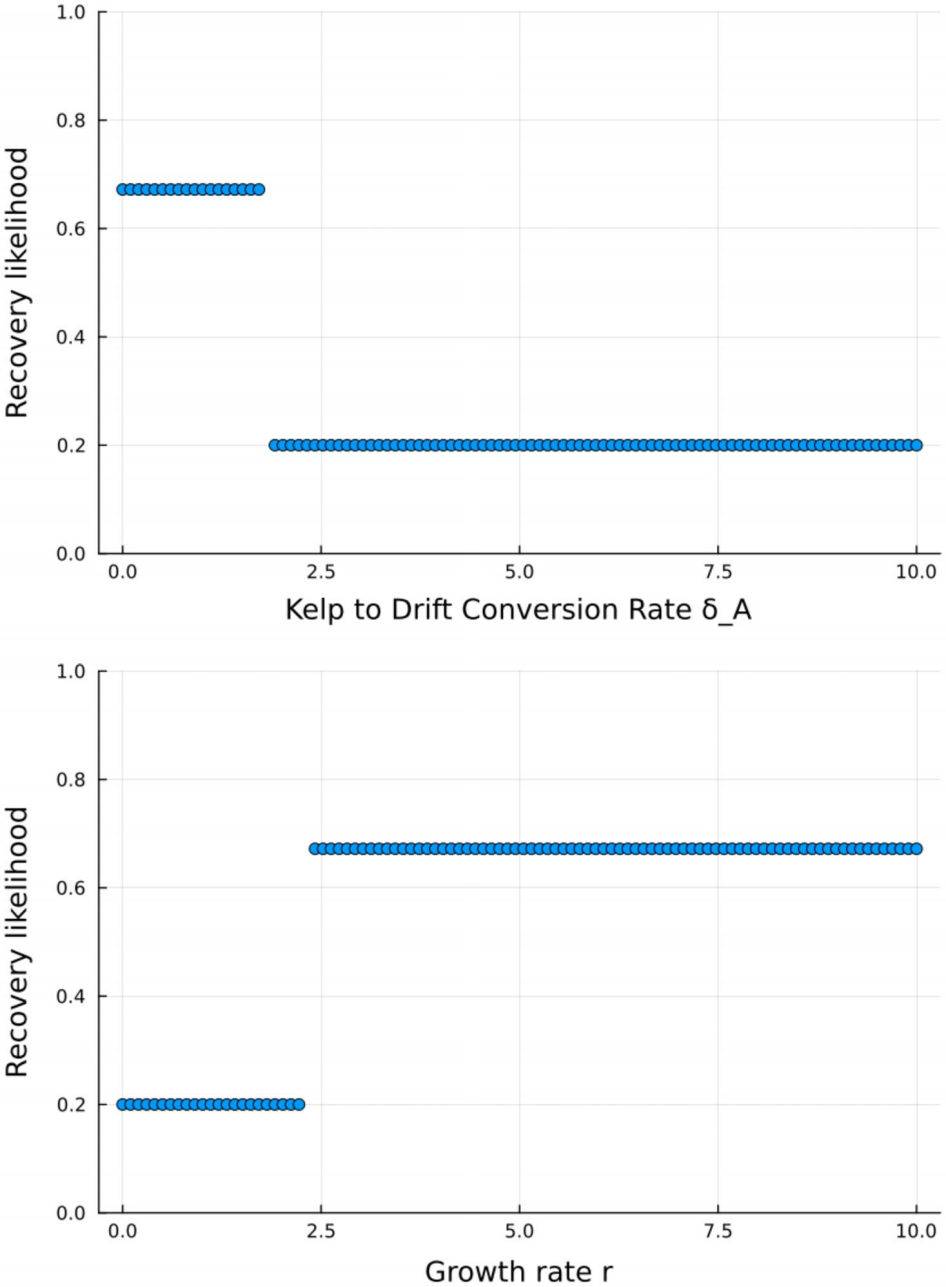
Trends of recovery likelihood as the top two most important parameters identified by the GSA (Figure 3) vary while the other parameters are fixed at their baseline value. See Table 1 for the baseline values of the other parameters.

If we vary the two most important parameters identified by the GSA of recovery likelihood with all other parameters at their baseline values, we observe that the recovery likelihood is a step function of each parameter with the breaking point being where kelp grows at the same rate as it produces drift kelp ($r=\delta_{A}$) (Figure 4). This is caused by the direct link between drift kelp production and kelp mortality in our model. A high per capita drift kelp production implies a high mortality rate, which then leads to a decreased recovery likelihood due to a reduced population replacement of kelp. This trend remains even if we look at a broader range of parameters by varying both kelp growth and drift kelp production (Appendix S1: Figure S1). When other parameters are varied, mainly the other parameters identified as most important by the GSA such as consumption conversion efficiencies and urchin preference for drift kelp (Figure 3), these parameters influence how likely it is for kelp to recover.

**FIGURE 5:**
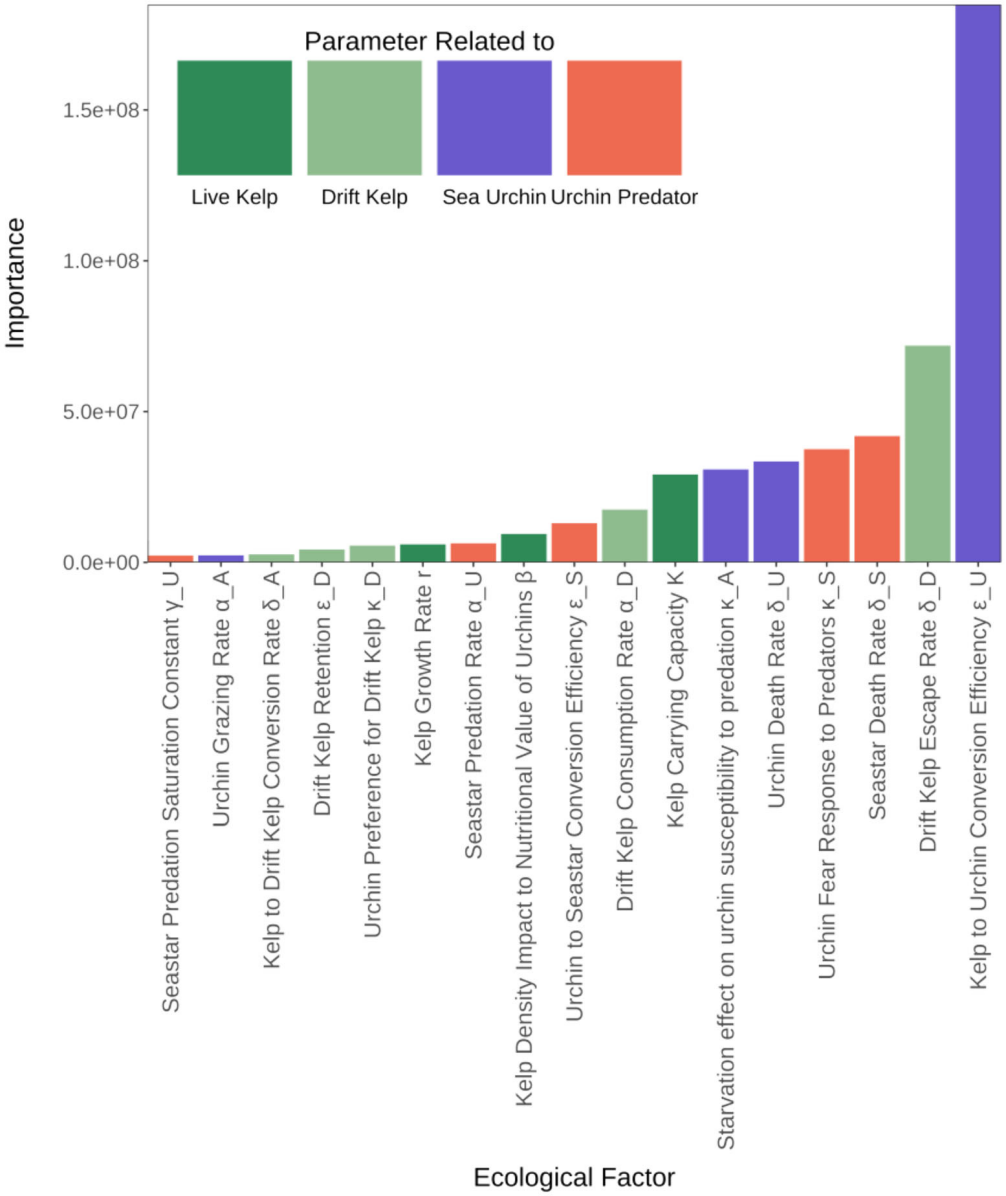
Importance ranking of the parameters of Model 2 from the global sensitivity analysis of the recovery rate of the kelp forest. See Table 1 for more detailed parameter definitions.

From our GSA, the recovery rate depends on a combination of parameters such as the kelp to urchin conversion efficiency ($\varepsilon_{U}$), drift kelp escape rate ($\delta_{D}$), and the fear response of urchins to predators ($\kappa_{S}$) (Figure 5). These results suggest that the recovery rate of the kelp forest is mainly determined by how fast urchins can actively graze live kelp stipes and how strong the feedbacks that limit their grazing activity (fear of predation and drift kelp preference) are.

**FIGURE 6:**
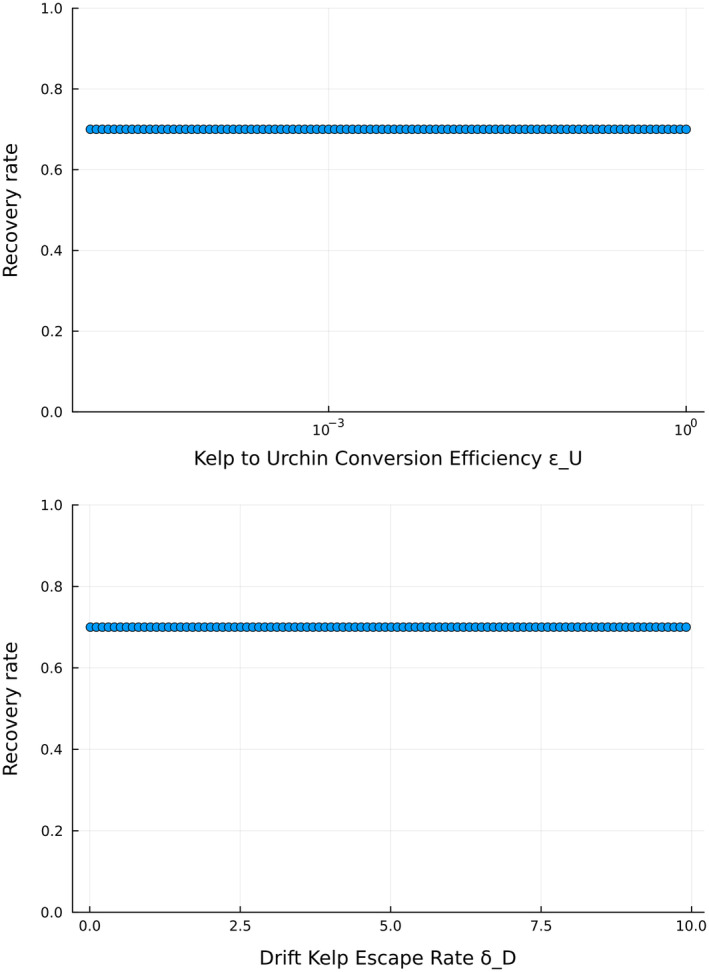
Trends of recovery rate as the top two most important parameters identified by the global sensitivity analysis (GSA) (Figure 5) vary while the other parameters are fixed at their baseline value. See Table 1 for the baseline values of the other parameters.

Under baseline parameter values, we do not observe a local effect of either the kelp to urchin conversion efficiency or the drift escape rate on the recovery rate (Figure 6). The recovery rate is affected by a combination of more than these two parameters explored in the GSA. Specifically, recovery rate noticeably increases for some parameter combinations at high urchin efficiency and drift kelp retention (low drift kelp escape rates) (Appendix S1: Figure S2).

**FIGURE 7:**
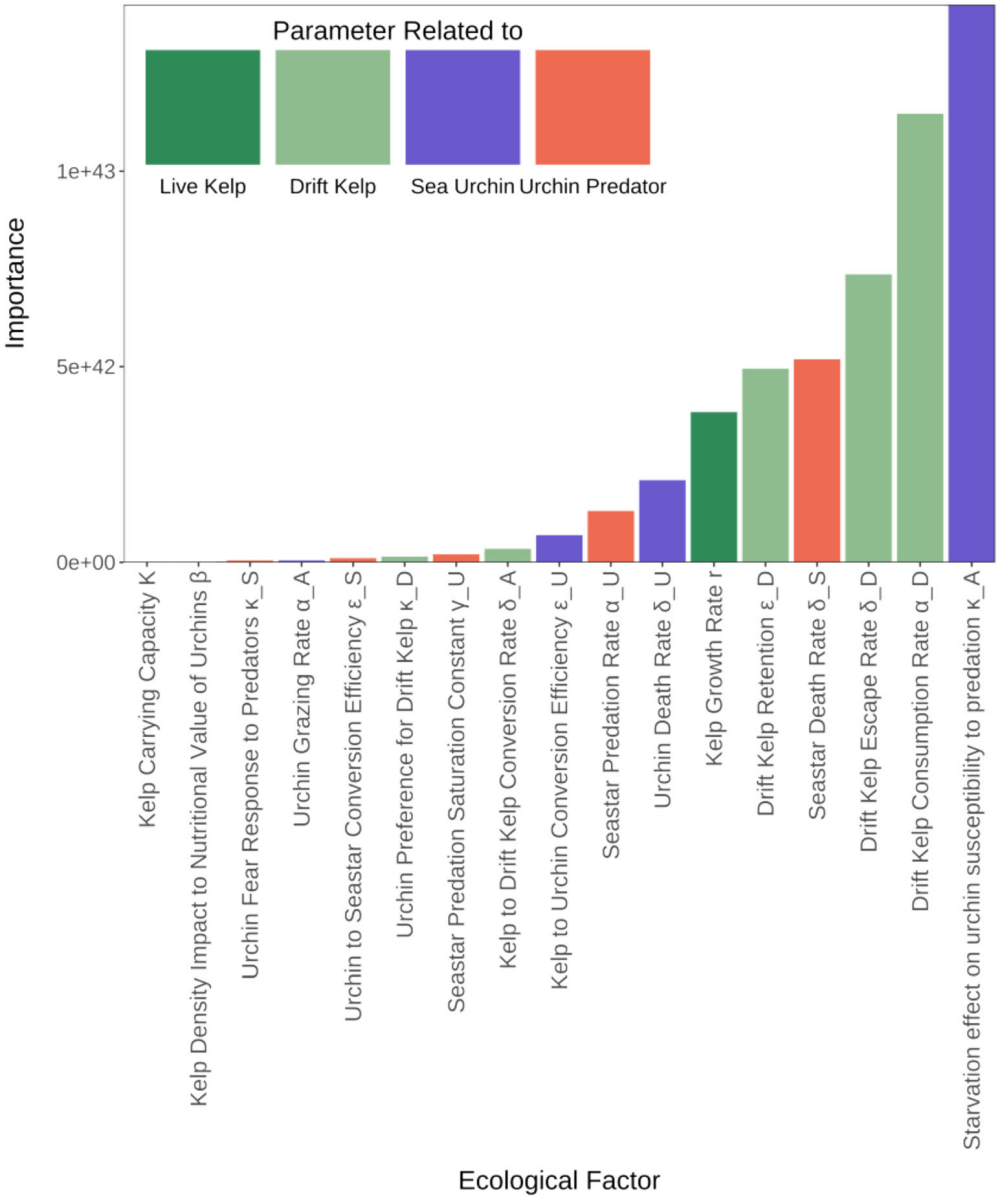
Importance ranking of the parameters of Model 2 from the global sensitivity analysis of the resistance to disturbance of the kelp forest. See Table 1 for more detailed parameter definitions.

Finally, the GSA shows that the kelp forest resistance depends the most on an interaction of the feedback produced by increased predation susceptibility with urchin starvation ($\kappa_{A}$) and drift kelp interactions, with the drift kelp consumption rate ($\alpha_{D}$) as the second most important parameter (Figure 7). This occurs because both the predator consumption of urchins (directly linked to urchin susceptibility to predation) and drift kelp production are deterrents of urchin active grazing, which means that as urchin active grazing decreases, a disturbed kelp will be less affected by direct grazing, which is the main factor that leads to the kelp decline and transition to urchin barrens.

**FIGURE 8:**
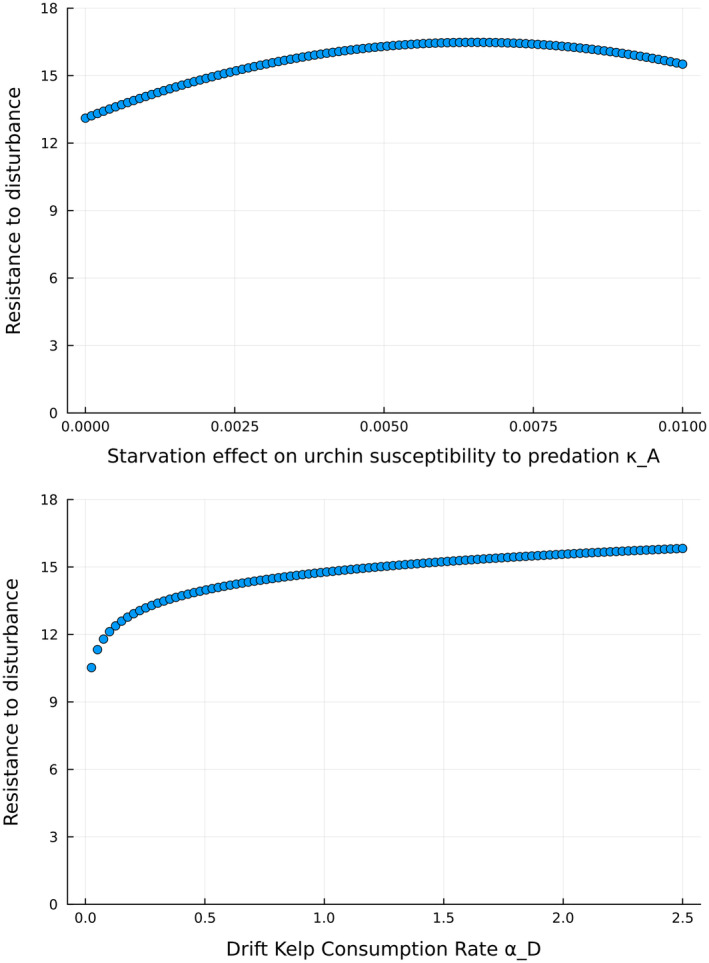
Trends of resistance to disturbance as the top two most important parameters identified by the global sensitivity analysis (GSA) (Figure 7) vary while the other parameters are fixed at their baseline value. See Table 1 for the baseline values of the other parameters.

This influence of urchin predation and grazing is supported by looking at the trends of resistance to disturbance as we vary the two parameters given baseline values for all other parameters (Figure 8). Here, we observe that the degree to which urchin starvation increases predation susceptibility has an impact on the resistance to disturbance, which slightly increases until it reaches a maximum, after which the resistance slowly starts to decline. This illustrates a possible trade‐off between a reduced grazing intensity by increased predation rate and a decreased nutritional value of starved urchins. In the case of the drift kelp consumption rate, an increasing consumption of drift kelp initially produces a rapid increase in the resistance to disturbance. This suggests that the kelp forest is more resistant when urchin active grazing is suppressed by higher drift preference. Varying all the parameters through a wider range does not show a clear trend, and in some scenarios, the kelp forest can be highly resistant to disturbance at low predation rates and vice versa, suggesting that resistance to disturbance is highly influenced by interactions with other parameters (Appendix S1: Figure S3).

## DISCUSSION

In this work, we have found that different resiliency metrics are driven by different ecological factors and interactions between them. For our resiliency metrics, we measured the proportion of conditions from which the system can return to the kelp forest state (recovery likelihood), the rate at which the system returns to this state (recovery rate), and how much disturbance is needed to transition the system from a kelp forest state to an urchin barren state (resistance to disturbance). We find that recovery likelihood depends the most on drift kelp through its relation to self‐replacement of the kelp population, which requires kelp growth to outpace loss to drift. Recovery rate is affected the most by grazing activity as it depends on a combination of drift kelp retention, which is directly linked to a combination of oceanographic factors such as seabed topography (Randell et al., 2022), turbulent mixing in the seabed (Layton et al., 2019), and fear of predation. Similarly, resistance to disturbance depends the most on a suppression in grazer density and grazing activity due to an increased drift kelp consumption rate and the degree to which urchin starvation increases predation susceptibility.

This difference in the influence of different ecological factors when resiliency is measured with different methods is a challenge of quantifying resiliency as noted by Quinlan et al. (2016) and Ingrisch and Bahn (2018). These studies postulate that using a single metric of resiliency limits a complete understanding of the drivers of ecosystem resiliency. To overcome this, both studies propose an assessment of resiliency using multiple metrics, as we have done here. By understanding how each different metric describes a different part of the ecosystem dynamics, we can understand the complementary roles of different restoration approaches connected to different ecological drivers in determining overall system resiliency.

In our Northern California kelp forest model system, the restoration approach currently underway is primarily urchin removal (Ward et al., 2022), with kelp reintroduction undergoing pilot trials; sunflower sea star reintroduction requires further research on feasibility. Our model suggests that each of these strategies can influence a different aspect of kelp forest recovery and persistence under future disturbance. Given its connection to kelp growth and drift kelp production, kelp reintroduction could particularly promote recovery likelihood and rate according to our model, which is in line with our previous modeling indicating its role in kelp forest recovery (Arroyo‐Esquivel et al., 2023). The role of at least one aspect of drift kelp processes (production and retention, which affect drift kelp availability to influence urchin grazing, as well as drift kelp consumption by urchins) in all three resilience metrics also reflects the potential efficacy of drift kelp supplementation, an emerging topic of investigation in California kelp forest systems, in achieving restoration goals. Urchin removal might also have an analogous impact to that of drift kelp preference in reducing the active grazing pressure that live kelp experiences; its potential role here echoes the efficacy found in other models and data (Arroyo‐Esquivel et al., 2023; Ward et al., 2022).

Our model further suggests that any future possibility of sea star reintroduction, which then promotes urchin consumption as it depends on urchin susceptibility and induces urchin fear responses, could particularly promote kelp forest resistance to future disturbance and recovery rate. Therefore, even if kelp forest recovery is achievable with the currently available tools of urchin removal and kelp reintroduction, development of sea star reintroduction approaches might still influence the persistence of any recovered kelp forests, especially given expectations of increased marine heat wave disturbances (Prochaska et al., 2023), an influence that requires a comprehensive analysis of resilience to identify.

The role of sea star reintroduction or recovery and its associated NCEs on the resistance to disturbance events is consistent with other analyses of food chains. In these analyses, the presence of top‐chain predators leads to a food chain that is more resistant to disturbances such as heat waves (Sentis et al., 2013) or nutrient enrichment (Llope et al., 2011). These results, in addition to our finding that sea star predation response to urchin susceptibility to predation is the main driver of resistance to disturbance, suggest that one of the main roles of a predator in determining ecosystem resiliency is to enhance a top‐down control over herbivores, which reduces the possibilities of overgrazing of the autotrophs in the population. Our analysis also shows that recovery rate is affected by suppression of grazers due to a fear effect induced by predators, which is consistent with that of other studies (Frank et al., 2011; Galloway et al., 2023). We did not find that this was the case in recovery likelihood, although other studies suggest that predation can play an important role in increasing recovery likelihood (Ripple & Beschta, 2003). It could be the case that predation plays an important role as well in recovery likelihood for our system. However, in our model, this is overshadowed by the effects of drift kelp preference by urchins. Note that the roles of urchin fear response and urchin susceptibility to predation effects in resistance and recovery rate echo the importance of NCEs in system resilience found elsewhere (Bestion et al., 2015; Sharp & Angelini, 2021).

It is important to note that the restoration interventions currently underway or planned in the Northern California kelp forest focus on changing the state of the system (e.g., urchin removal reduces the density of urchins present, or kelp outplanting increases kelp density) with underlying parameters remaining unchanged. Any restoration plan that focused on permanently changing the parameters of the system (such as continual urchin removal to increase the natural death rate of urchins) could lead to a “conservation‐reliant” system where the state of the system would not be able to be sustained in the absence of human intervention (Scott et al., 2005). However, temporarily engaging in some interventions, such as those mentioned in the previous paragraph, could alter these parameters during crucial post‐disturbance or recovery phase periods.

While each resiliency metric has unique drivers, our model suggests that the overarching mechanism that leads to a more resilient kelp forest is the preference of drift kelp by urchins. This behavior has been shown to be one of the main factors suppressing the grazing activity of urchins as field observations have shown that urchins tend to hide on crevices in the presence of drift kelp (Dayton, 1985; Kriegisch et al., 2019). This by itself hints to an increased resiliency of the kelp forest due to the preference of urchins to drift kelp. However, its ubiquity through all resiliency metrics bolsters its role as a particularly important factor.

Through this work, we based our model parameters on data from tank experiments using the sunflower sea star as an urchin predator, which was found to prey more on starved urchins than fed urchins (Galloway et al., 2023). Another predator that has been historically present in the northern coast of California is the sea otter *E. lutris*, which has been locally extinct since the mid‐1800s. Sea otters avoid hunting starving sea urchins with low gonad content and prefer other types of prey (Smith et al., 2021). However, otters do consume barren urchins when they are at a particularly high density, as observed in the Aleutian Islands (Stewart & Konar, 2012). Furthermore, urchins in a recovered forest (the state where urchin consumption has the most influence in our model as that is where resistance is a relevant resilience metric) will not be barren urchins and therefore are more likely to be an attractive prey for sea otters. In addition, predation‐associated dynamics being the most important factor in determining resistance to perturbations like extreme weather events is consistent with the lower impact of the 2015–2016 marine heat wave in Central California, where a sea otter population exists, compared with Northern California, where, in the absence of sunflower sea stars, no functional predator population is currently present (Beas‐Luna et al., 2020). Therefore, like sea stars, the effect of sea otters might be the greatest for resistance of a kelp‐dominated state as compared with other aspects of resiliency.

In addition to our choice of predator, we have made other assumptions to ensure that we have the simplest model relevant to our central question. Our model focused on the interactions of the kelp–purple urchin–predator food chain. However, the temperate rocky reef of the northern coast of California is home to other grazers of kelp, as well as prey for the sunflower sea star, such as red abalone, red urchins, and some crustaceans (Springer et al., 2010). Including these species into our model would probably reduce the relative importance of drift kelp and predation rate of urchins by predators and allow other parameters to gain importance. However, given the evidence from other studies within and beyond the kelp forest referenced throughout this paper, we would not expect other ecological drivers to become more important than the two main drivers found in our work. Similarly, if our food chain included two urchin predators (e.g., sea otters as well as sunflower sea stars), we would not expect the competition of these predators for urchins to significantly change these effects, as trophic redundancy has been shown to promote kelp resiliency in the southern coast of California (Eisaguirre et al., 2020). This is further supported when considering that sea otters tend to predate on different urchin sizes than sea stars, making each predator complement each other (Burt et al., 2018).

A more potentially impactful limitation of our model is our representation of producer dynamics. Bull kelp is a primarily annual species, where most kelp stipes grow during spring and summer and decline through fall and winter (Maxell & Miller, 1996). This seasonal, discrete‐time nature of bull kelp dynamics is not incorporated in this model and has the potential to reduce the range of growth rate values explored in this work. Furthermore, bull kelp is one of multiple macroalgal species than can have a complex interplay of positive and negative interactions (Liu & Gaines, 2022). Accounting for the interactions of kelp with other species could allow the exploration of the roles of diversity and redundancy as well as feedback loops in affecting different aspects of resiliency. This is an open question that will be explored in a future work.

In conclusion, we find that a comprehensive evaluation of resilience with multiple metrics reveals complementary roles of different ecological processes and therefore the complementary roles of multiple restoration interventions that might differentially affect these processes. The difference in the drivers for resistance in particular, as compared with the drivers of recovery likelihood and recovery rate, suggests that restoration actions that might successfully recover a target ecosystem state might still leave it vulnerable to future disturbance. Therefore, further investigation into the drivers of multiple aspects of resilience in systems beyond California kelp forests can help inform a restoration goal of protecting ecosystem structure and function given the ongoing global change (Harris et al., 2006).

## CONFLICT OF INTEREST STATEMENT

The authors declare no conflicts of interest.

## Supporting information


Appendix S1.

## Data Availability

Data for drift kelp preference and consumption rates of live kelp and drift kelp (Randell & Novak, 2024) are available in Zenodo at https://doi.org/10.5281/zenodo.13869602. Data for urchin predation rates and the effects of urchin starvation on predator predation (Galloway et al., 2023) are available in Dryad at https://doi.org/10.5061/dryad.zcrjdfngr. Data for the effects of fear of predation (Whippo, 2024) are available in Zenodo at https://doi.org/10.5281/zenodo.7850793. Code (Arroyo‐Esquivel, 2024) is available in Zenodo at https://doi.org/10.5281/zenodo.13499903.
