# Supplementary material for: Multiple resiliency metrics reveal complementary drivers of ecosystem persistence: An application to kelp forest systems
Source: Ecology. 2024 Oct 27;105(12):e4453. doi: 10.1002/ecy.4453 (PMC11610656; doi:10.1002/ecy.4453)
Supplement: Supplementary file 1 — Appendix S1. [file ECY-105-e4453-s001.pdf]

Supporting Information

Multiple resiliency metrics reveal complementary drivers of ecosystem persistence: An application to kelp forest systems

Jorge Arroyo-Esquivel, Riley Adams, Sarah Gravem, Ross Whippo, Zachary Randell, Jason Hodin, Aaron Galloway, Brian Gaylord, and Marissa L. Baskett

Ecological Applications

Appendix S1

Sampled measurements of resiliency metrics for a wider combination of parameters

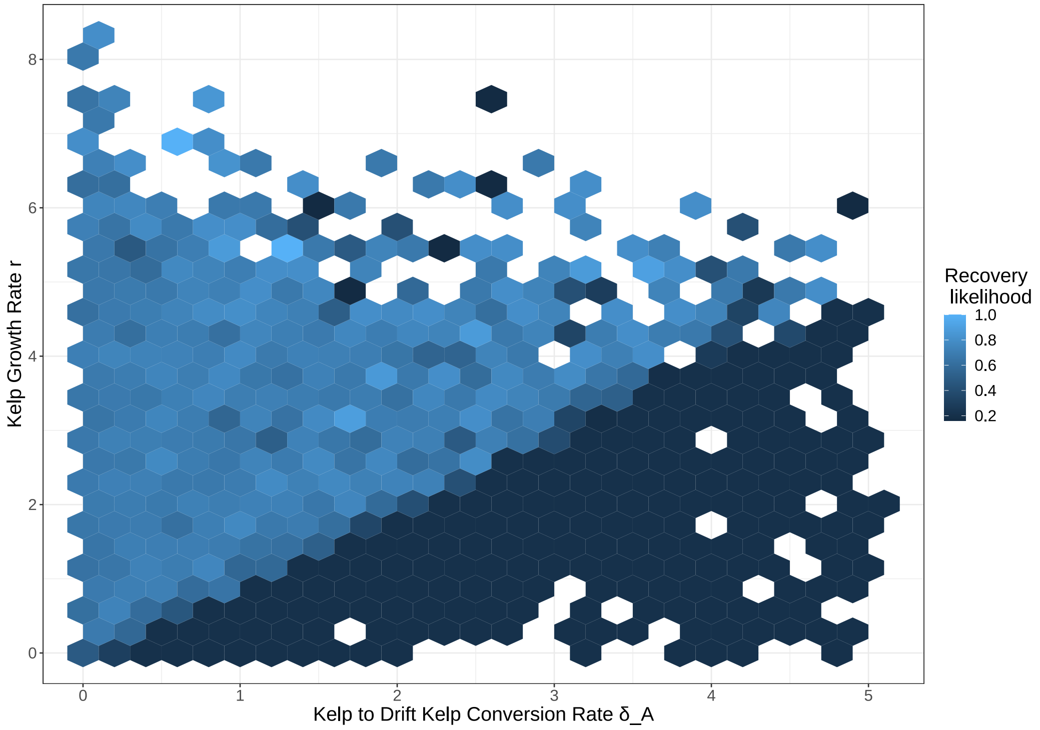

Figure S1: Sampled measurements of recovery likelihood for different combinations of the parameters of the model sampled from their distribution of values explored (see Table 1). Each hexagon represents the mean of the metric for the sampled parameter values found inside the hexagon.

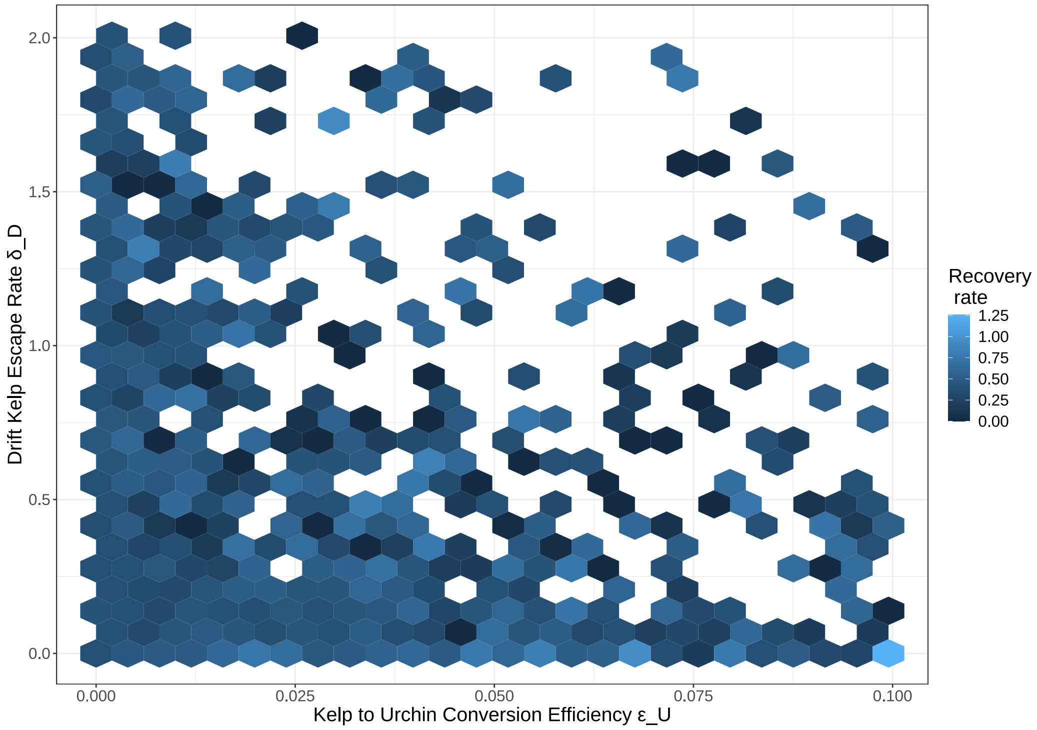

Figure S2: Sampled measurements of recovery rate for different combinations of the parameters of the model sampled from their distribution of values explored (see Table 1). Each hexagon represents the mean of the metric for the sampled parameter values found inside the hexagon.

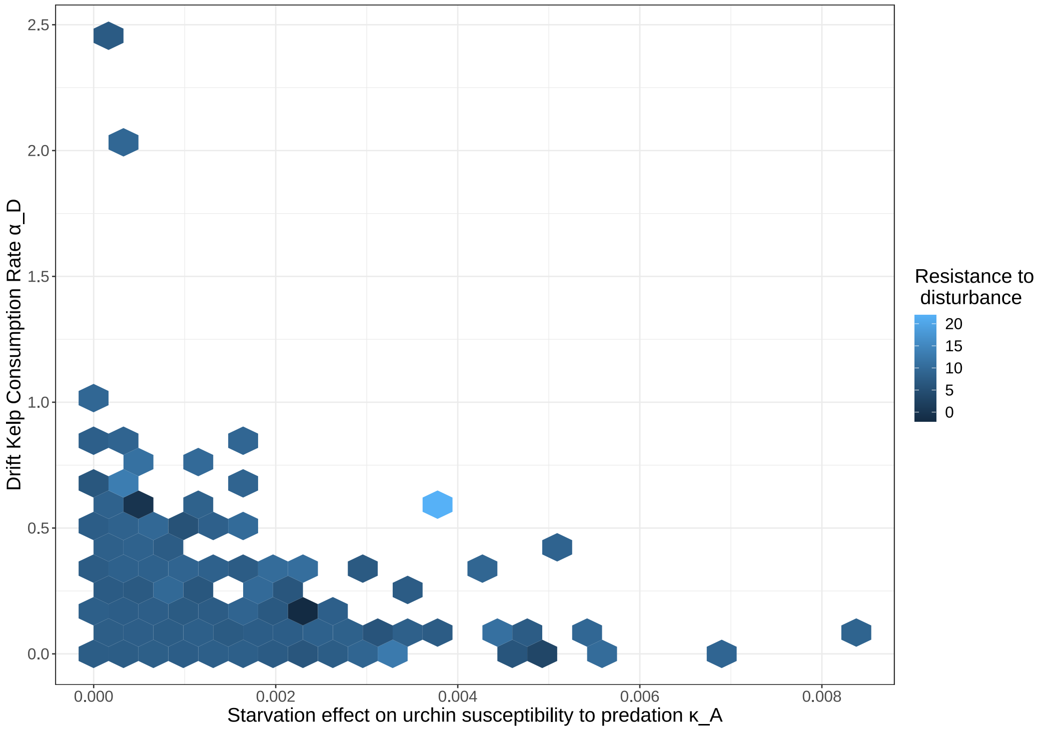

Figure S3: Sampled measurements of resistance to disturbance for different combinations of the parameters of the model sampled from their distribution of values explored (see Table 1). Each hexagon represents the mean of the metric for the sampled parameter values found inside the hexagon.
